# Whispers in the Lungs: Small Extracellular Vesicles in Lung Cancer and COPD Crosstalk

**DOI:** 10.3390/cancers17101612

**Published:** 2025-05-09

**Authors:** Yetemwork Aleka, Fantahun Biadglegne, Ulrich Sack

**Affiliations:** 1 Institute of Clinical Immunology, Faculty of Medicine, Leipzig University, 04103 Leipzig, Germany; 2 Department of Immunology, College of Medicine and Health Science, University of Gondar, Gondar P.O. Box 196, Ethiopia; 3 College of Medicine and Health Sciences, Bahir Dar University, Bahir Dar P.O. Box 79, Ethiopia; 4 Clinical and Translational Research Unit, School of Medicine, Stanford University, 800 Welch Rd, Palo Alto, CA 94304, USA

**Keywords:** small extracellular vesicles, lung cancer, COPD, biomarkers

## Abstract

Lung cancer is one of the deadliest forms of cancer. Its prognosis becomes even worse when it co-occurs with other diseases, such as chronic obstructive pulmonary disease (COPD). Both illnesses have numerous shared risk factors, including the use of tobacco smoke, and have similar underlying mechanisms like long-term inflammation. There are some other less studied but equally important molecules, like small extracellular vesicles (sEVs), that have been shown to mediate effective communication at the cellular level and may affect the progression of a disease or cause resistance to therapies. In sEVs from lung cancer tumors, there are onco-proteins (e.g., tumor initiator EGFR mutations, onco-miR, miR-21), while in sEVs from patients with COPD, there are pro-inflammatory cytokines like IL-6 and TNF-α that enhance airway inflammation. These potential biomarkers of sEVs from chronic lung disease have great value in defense against emerging health problems; however, limitations in sample extraction and analysis are obstacles that hinder clinical enhanced applicability. This review focuses on sEV-derived biomarkers in lung cancer and COPD for diagnostic, prognostic, and therapeutic monitoring purposes. To make these molecules more useful in real-life therapy and determine their signature’s role, further investigation with a high-scale study is necessary.

## Simple Summary

Lung cancer is a very serious disease, and it becomes worse when combined with chronic obstructive pulmonary disease [COPD], another chronic lung illness. Both are linked to smoking and long-term lung inflammation. Scientists are now focusing on tiny particles called small extracellular vesicles (sEVs), which are released by cells and carry important molecules. In lung cancer, sEVs often carry cancer-related signals, while in COPD, they carry inflammation-related ones. These signals could help doctors to diagnose and monitor the diseases more accurately. However, collecting and studying sEVs is still difficult, which limits their use in clinics. This review looks at the potential of sEVs as helpful tools in treating lung cancer and COPD and highlights the need for more research.

## 1. Introduction

**Figure 1:**
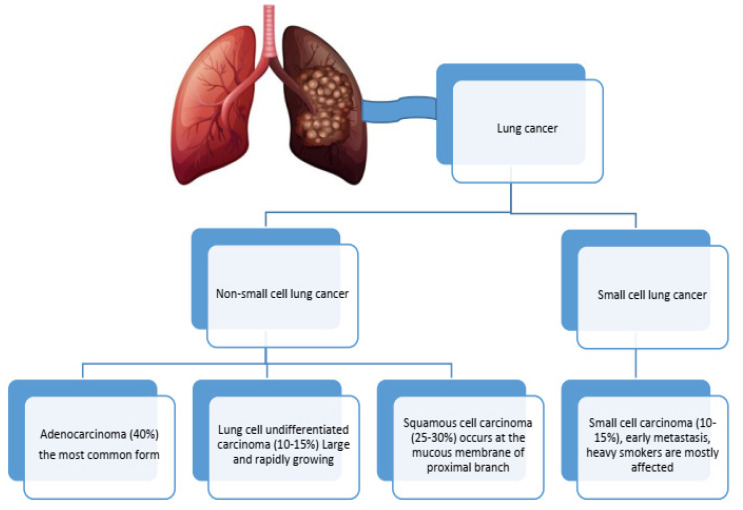
The most common types of lung cancer.

As time elapses, the list of people dying from lung cancer continues to increase, owing to its rank as the second most fatal cancer type across the globe, with 2.5 million new cases and 1.8 million deaths annually. The development of lung cancer in conjunction with chronic obstructive pulmonary disease (COPD) differs by gender, age, and geographical location. The risk is approximately twofold for male COPD patients as opposed to females (5.09% versus 2.52%). However, the risk continues to grow with age, with more than 10% of younger lung cancer patients also having COPD. Geographically, the Western Pacific has the highest prevalence of COPD at 7.78%, followed by 3.25% in the Americas and 3.21% in Europe [1]. Smoking is the primary risk factor, alongside pollution and pre-existing lung conditions like COPD, which also contribute to high mortality rates. Late-stage diagnosis significantly affects survival, with 77% of cases being diagnosed at advanced stages [2]. Diagnosis involves using imaging techniques and biopsies to classify lung cancer into non-small-cell lung cancer (NSCLC) and small-cell lung cancer (SCLC) (Figure 1). Treatment varies by type and stage, including surgery [2], radiation, chemotherapy [3], and targeted therapies [4,5] based on genetic mutations. Recent advances in medicine, particularly using the immune system in treatment through immunotherapy [6], have shown immense possibility, especially concerning severe NSCLC cases. To improve the condition of the afflicted, the expansion of screening with the employment of new innovative biomarkers that aid in improving diagnosis and screening approaches is required. Efforts towards early detection screening have emerged to help lung cancer patients immensely. This is important in ensuring better outcomes for managing affected individuals. Scientists are searching for new biomarkers to aid in early lung cancer diagnosis and determine outcome prediction with or without other respiratory disease comorbidity [4,7,8,9,10]. The goal of this narrative review is to identify the roles, functions, and diagnostic or prognostic value of small EVs released from lung cancer or COPD patients using different original articles from peer-reviewed publishers such as Elsevier, Science Direct, PubMed, Scopus, Web of Science, and Google Scholar. It also analyzes the potential difficulties in distinguishing accurate small extracellular vesicle biomarkers considering the presence of comorbidities. We address the issues of interpreting biomarkers with concurrent pathologies and highlight important factors needed to refine accuracy in multi-faceted clinical situations.

### Dual Battles: Immune Dynamics in COPD and Lung Cancer Progression

The immune system, which includes T cells, B cells, NK cells, macrophages, and dendritic cells, is essential in defending the body from cancerous cells. With lung cancer, the tumor microenvironment (TME) is a complex ecosystem that not only fosters tumor development but also shields it from immune response [11]. Cancer cells commonly use tactics to escape immune surveillance and strike, for instance, the downregulation of molecules responsible for antigen presentation [12], immunosuppressive cytokine secretion, inhibitory receptor expression, and recruitment of immunosuppressive cell types [13].

Furthermore, COPD is defined by persistent inflammation and constriction of the airways following the inhalation of harmful particulates or gases, especially cigarette smoke [14]. The response of the immune system is quite complicated and often overlaps with non-small-cell lung cancer, which is associated with a higher likelihood of lung cancer. Pro-inflammatory cytokines are released by macrophages and neutrophils in COPD, leading to tissue damage and mucus hypersecretion [15]. The cells that include Th1 and Th17 subsets activate macrophages and worsen inflammation. On the other hand, plasma cells differentiated from B cells are also active in inflammation [16]. The increased levels of inflammatory proteins and chemokines critically damage airway, cause inflammation and modify the environment. Tissue damage from oxidative balance in COPD is detrimental to the cells and increases inflammation. There are often such changes with persistent inflammation to the structure of the airways, which leads to further issues with the limitation of airflow and respiratory problems. This means that the region is subject to hyperinflammation, which may lead to a more persistent infection.

A combination of these two diseases can severely suppress immunity in the affected individual [17], making them experience elevated systemic oxidative stress and diminished levels of antioxidants and inflammatory cells [18]. Sustained lung inflammation due to irritants like cigarette smoke or neoplastic cells can be the cause of the priming of the immune system, shifting to the inflammatory state alongside tissue damage and further inflammation. COPD coupled with lung cancer can also increase the incidence and severity of infection due to impaired lung function and immunity while enhancing the chances of altering treatment selection and changing treatment response towards lung cancer. These two diseases can also lead to the compromising of the immune system, causing a shift towards a pro-inflammatory response and favoring tumor cell evasion [19]. The coexistence of these two conditions can also affect the selection and efficacy of lung cancer treatments, as well as increase susceptibility to respiratory infections due to compromised lung function and weakened immune response [20].

Therefore, early diagnosis and prognosis of treatment and its efficacy biomarkers is significant, as these biomolecules are expected to be accurate and precise. Recently, attention has shifted to extracellular vesicles (EVs) such as small EVs for their potential use in diagnostic and monitoring treatment purposes [10]. These biomolecules are noteworthy due to their small dimensions and originate from a unique process of production, namely an endocytic process, where they are substantially produced from parent cells. In the diseases of lung cancer and COPD, small EVs are known to participate in progression, immune modulation, and tissue remodeling, which goes beyond the activities of cells and includes tissue repair and fibrosis [21].

## 2. Small EVs as a Hidden Language

EVs are membrane-bound structures released by cells into bodily fluids that facilitate intercellular communication by transporting proteins, lipids, nucleic acids, and metabolites [22]. Varieties of these messengers are further explained in Table 1 and include small EVs (30–150 nm, located in multivesicular bodies) [23], microvesicles (100–1000 nm, from plasma membrane budding), and apoptotic bodies (500–2000 nm, released during cell death). EVs mediate development, physiological processes, and the regulation of immunity and have future potential in scientific investigation and medicine delivery. There are a few other subdivisions of EVs that are known, but for their indefinite overlap in size and biogenesis pathways, they are mostly included in one of the three main EV categories, as mentioned above. Certain EVs, however, enclose distinct molecular identities or functional roles in living systems, but within the context of current understanding, their classification will remain primarily based on their size. More studies must be conducted on the biogenesis, molecular composition, and functional attributes for EVs to aid in determining applicable classification strategies.

## 3. From Cells to Circulation

Small EVs facilitate intercellular communication, maintaining cellular balance and immune function [27]. They are secreted ubiquitously across cell types including γδ T lymphocytes, which demonstrate potential as cancer immune-therapeutics. Tumor-derived EVs function as antigen carriers, while immune cell-derived vesicles exhibit intrinsic antineoplastic activity via bioactive molecular cargo [28]. They promote cancer progression through angiogenesis [29], oncogenic molecule transfer, and metastatic site preparation [30]. EVs originate from tumor cells (promoting growth, angiogenesis, metastasis, immune evasion), immune cells (modulating anti-cancer responses), and respiratory cells. In lung cancer, tumor-derived EVs facilitate progression and metastasis [31]. During COPD, inflammation causes the immune system to be over activated and release pro-inflammatory mediators that damage tissues and remodel airways [32], while cigarette smoke-damaged epithelial cells release inflammatory EVs [14,33,34]. sEVs from patients suffering from COPD exhibited significantly higher levels of IL-1β, TNF-α, MMPs [35], and dysregulated miRNAs like miR-21 [34]. In a clinical setting, sEVs can be used as diagnostic and prognostic biomarkers for the detection of the disease in its early stages [36,37], disease monitoring [38,39], and even as therapeutic agents through the modulation of the sEV-mediated routes [40].

EV autoantibodies have been also identified as potential biomarkers in lung cancer, complementing their previously established role of EVs in biomarker identification. This finding expands the repertoire of molecular signatures that may facilitate the early detection and characterization of lung malignancies. The presence of these autoantibodies in patient serum provides an additional dimension to the multi-faceted approach of cancer diagnostics, potentially enhancing sensitivity and specificity when used in conjunction with EV-derived biomarkers [41].

## 4. From Sample to Signal: The Challenge of Isolation and Characterization

There is a significant lack of consensus regarding standardized protocols for EV isolation and characterization among researchers, representing a critical bottleneck in comparative studies of EVs with other molecular entities (Table 2). Despite numerous techniques being available, including ultracentrifugation [42], density gradient approaches [43], polymer-based precipitation [44], and size exclusion chromatography [45], methodological variations yield heterogeneous EV populations with differing purity profiles.

Similarly, characterization methods ranging from NTA [46], TEM [47], and flow cytometry to advanced techniques like fluorescence NTA [48] and ExoArc-SEC coupling [49] produce inconsistent results across laboratories. This methodological inconsistency impedes biomarker discovery efforts in lung cancer and COPD, where protein analysis workflows encompassing extraction, quantification [23], and profiling require rigorous standardization to establish clinically relevant markers [50].

Additional characterization includes lipid assays [51], functional assays, RNA expression analysis via qPCR [52], and Surface Plasmon Resonance (SPR) for binding kinetics [53]. On the other hand, most peripheral EVs are platelet-derived, creating significant challenges in developing EV-based diagnostic and prognostic markers. Platelets release abundant EVs during both normal physiology and sample processing, generating background noise that masks disease-specific signals. For reliable EV biomarker research, effective platelet removal protocols must be implemented [54]. Consequently, the field urgently requires guidelines establishing reproducible isolation and characterization protocols to facilitate meaningful cross-study comparisons and accelerate EV-based clinical applications.

**Table 2:** EV isolation and characterization methods.

Method		Advantage	Disadvantage	Reference
Isolation	Ultracentrifugation	Large volumes processing	Time-consuming, costly equipment, non-EV contamination risk	[42]
	Density graident centrifugatin	Higher purity	Labor and time intensive	[43]
	Immunoaffinity capture	High specificity	Potentia.antibody carryover, expensive	[44]
	Polymer precipitation	Simple	Contaminants, co-precipitation	
	Size exclusion chromatography	Gentle on EVs	Limited sample volume, EV dilution	[45]
	Microfluidic device	Rapid	Device.fabrication complexity; potential for clogging	[49]
Characterization	NTA	Provides size distribution and concentration, relatively quick	Limited sensitivity for small EVs, affected by sample purity	[46]
	Western blotting	Confirms presence of EVs marker	Labor and time consuming	[50]
	Flowcytometry	Multiparametric analysis	Limited sensitivity for small EVs	[50]
	TEM	Detailed, structural information visually	potential artifacts, not quantitative	[47]
	Dynamic light scattering	Quick	Less accurate for polydisperse samples	[55]
	SPR	Measures EV binding affinity	Complex data interpretation	[53]

EVs—extracellular vesicles; NTA—nano-particle tracking analysis; TEM—transmission electron microscope.

## 5. Unlocking Cargos

Herein, we define small EVs possessing unique bioactive characteristics that allow the modulation of several cellular activities. The comprehensive protein composition of small EVs involves an array of proteins related to membrane transport, antigen capturing, and cell communication. Additionally, small EVs carry different species of RNAs such as messenger RNA (mRNA), microRNA (miRNA), and long non-coding RNA (lncRNA) that alter the expression of genes in each target cell. Furthermore, the small EVs comprise lipids to improve their structural stability due to their ability to bind with the cells of interest [56].

The identified biomarkers listed in Table 3 are diagnostically noted to be present in the context of lung cancer. However, the coexistence of these biomarkers may allow us to reasonably assume alterations to the downregulation or overexpression modifier. Their expression will be based on their relationship with lung cancer pathology; however, there is room for, and need for, further examination.

## 6. Bridging the Dual Treats: Expression Role in Comorbid Lung Cancer and COPD

While identifying disease-specific sEV markers in comorbid conditions remains difficult, some markers have been identified for various pathological states. The dominant pan-EV markers are tetraspanins (CD9, CD63, CD81), endosomal sorting complex required for transport (ESCR) components (TSG101, ALIX), and membrane proteins (flotillin-1, annexins). Furthermore, the conserved cargos across diverse cell-derived EVs are heat shock proteins (HSP70, HSP90) and cytoskeletal components (actin, tubulin), as demonstrated by the comparative analysis presented in Table 4. The presence of these markers facilitates the isolation and characterization of EVs, but their widespread presence is counterproductive when attempting to identify disease-specific vesicle populations. This highlights the need for further refined approaches to analyze the EV signatures of distinct diseases, especially with more complex pathological cases that have comorbidities [90].

## 7. Concluding the Promise of sEVs

The field of lung cancer sEV markers is developing quickly and has considerable prognostic and therapeutic prospects. The discovery of sEV-associated proteins, microRNA (miRNA), and DNA mutations elements enable non-invasive early diagnosis and treatment decisions. Yet, the application of these strategies in clinical practice needs to address standardization, secure large clinical studies, and resolve ethical issues.

The integration of sEV markers into general medical practice may improve the prognosis and quality of life of patients suffering both from lung cancer and COPD. We suggest the study of sEV markers during co-infections of COPD, allergies, and other inflammatory diseases to gain better insights and formulate new diagnostic and therapeutic strategies. To conclude, though the sEV markers in question are extremely promising, their clinical application is bound by the need for extensive proof of concept with supporting data. Their adoption and integration will reshape lung cancer management and care alongside other related diseases, ultimately improving patients’ health and quality of life. 

## Figures and Tables

**Table 1:** Classifications of EVs.

EV Types	Size	Density (g/mL)	Markers	Biogenesis and Secretion	Pathway	Reference
Small EVs	30–150 nm	1.12–1.19	CD9, CD63, CD81	Inward budding of the late endosome lumen and fusion with the plasma membrane	ESCRET-dependent and Tetraspanin-dependent	[24]
Microvesicles	100–1000 nm	1.12–1.21	CD40-ligand, se-lectin, flotillin-2, annexin 1	Outward budding and fission of the plasma membrane	Ca^2+^-dependent and Stimuli-and-cell-dependent	[25]
Apoptotic bodies	1–4 µm	1.16–1.28	AnexinV, DNA, histones, phosphatidylserine	Plasma membrane blebs of cells undergoing apoptosis	Apoptosis-related	[26]

EV—extracellular vesicles; ESCRET—endosomal sorting complex required for transport; CD—cluster of differentiation; nm—nanometer; µm—micrometer.

**Table 3:** Genetic, protein, and lipid biomarkers of small EVs that were identified during lung cancer infection.

Marker	Specific Types	Measurement	Clinical Significance	Reference
Genetic markers I. MicroRNA	miR-21	Overexpression	poor prognosis, invasion, and tumor growth	[57]
	Let-7	Downregulated	tumor aggressiveness	[58]
	miR-155	Upregulation	invasion	[59]
	miR-375-P	Low expression	metastasis	[22]
	miR-210p	Overexpression	poor prognosis	[9]
	miR-126	Downregulation	tumor progress	[60]
	miR 200	Overexpression	tumor metastasis, drug resistance, and immune modulation	[61]
	miR-199a-5p	Low expression	metastasis	[35]
	mir 146a-5p	Over expression	metastasis	[62]
	miR-320	Low expression	treatment resistance	[63]
	miR-486-5p	Lower expression	advanced stages and poor prognosis	[64]
II. Long non-coding RNAs (lncRNAs):	Metastasis-Associated Lung Adenocarcinoma Transcript1 (MALAT1)	Overexpression	poor prognosis and invasion	[65]
	HOX Transcript Antisense Intergenic RNA (HOTAIR)	Dysregulated Overexpression	metastasis and poor prognosis.	[66]
	RP5-977B1	Elevated expression	tumor growth, proliferation	[67]
	Long intergenic non-coding RNA 917 (LINC00917)	Elevated levels	metastasis	[68]
III. mRNAs (messenger RNAs) and DNA mutations	Epidermal Growth Factor Receptor (EGFR)	Mutations	progression	[69]
	ALK (Anaplastic Lymphoma Kinase), BRAF, MET	Gene rearrangements	tumor shrinkage or stable disease	[70]
	Kirsten Rat Sarcoma Viral Oncogene-Homolog (KRAS)	Mutations	poor prognosis and limited response to certain, targeted therapies.	[70]
	ROS Proto-Oncogene 1, Receptor Tyrosine Kinase (ROS1)	Gene rearrangements	metastasis NSCLC	[70]
	Programmed Death-Ligand 1 (PD-L1) and tPDL-1	Overexpression	immune evasion	[71]
	Kirsten Rat Sarcoma Viral Oncogene.Homolog (KRAS)	Mutations	poorer prognosis	[72]
	V-Raf Murine Sarcoma Viral Oncogene Homolog B (BRAF)	Mutations and over-expression	cancer development and progression	[38]
	Tumor Protein P53 (TP53)	Mutations	aggressive phenotype and poor prognosis	[38]
	Rearranged-During Transfection (RET)	RET fusions	metastasis	[70]
Proteins	Heat Shock Protein 70 (HSP70, HSP90)	Elevated expression	poor prognosis, metastasis	[73]
	Carcino-embryonic Antigen (CEA)	Elevated expression	poor survival	[27]
	ALIX	Elevated level	metastasis	[74]
	CD151	Elevated level	tumor growth	[75]
	CD63, CD133	Elevated level	metastasis and immune invasion	[76,77]
	CD105	Elevated levels	Metastasis	[57]
	EpCAM (Epithelial Cell Adhesion Molecule)	Upregulation	tumoral transformation	[78]
	Tetraspanin proteins CD9, CD63, and CD81	Elevated dysregulation	cancer cell growth, drug resistance, metastasis, stemness,	[79]
	EGFR.(Epidermal GrowthFactor Receptor)	Overexpression	Poor survival	[71]
	MUC1 (Mucin 1)	Overexpression	cancer development and metastasis	[80]
	Tumor Susceptibility Gene 101 (TSG101)	Mutation	Metastasis	[81]
	CD5L	Elevated expression	cancer progression	[82]
	IL-6, IL-8	Elevated expression	high-inflammation, chemoresistance, immune evasion	[83]
Lipids	Cholesterol Ester	Altered levels	disease progression	[84]
	High-Density Lipoprotein (HDL) and Low-Density Lipoprotein (LDL)	Low levels	Metastasis	[85]
	Sphingomyelin (SM)	High levels	metastasis/angiogenesis	[86]
	Phosphatidylserine (PS)	Alterations	cancer progression	[87]
	Phosphatidylethanolamine (PE)	Overexpression	Poor prognosis	[88]
	Phosphatidylcholine (PC)	Overexpression	Poor prognosis	[85]
	phosphoinositide 3-kinase (PIK3CA):	High expression	lung cancer progression.	[85]
	Lysophosphatidylcholine (LPC)	Low expression	cancer progression	[85]
	Ceramide-Synthase Enzymes	Dysregulation	Progression.	[89]
	C16:0 Ceramide	High expression	cancer progression and metastasis	[86]
	C18:0 Ceramide	Low expression	cancer progression	[86]

miR—microRNA.

**Table 4:** Common EV markers during lung cancer and COPD.

Marker	Expression	Role in Comorbidity	Reference
CD 9	Dysregulated	altered cell adhesion and metastasis	[91]
CD 63	Elevated	immune evasion, chronic inflammation,	[91]
CD 81	Increased	tumor progression	[91]
TSG 101	upregulated	tumor progression	[35]
ALIX	Increased	tumor cell survival and COPD-related lung remodeling	[32]
PD-L1	Upregulated	immune evasion	[90]
HSP70 and HSP90	Elevated	tumor survival	[90]
miR-21	overexpressed	tumor progression	[35]
miR-155	Overexpressed	immune suppression	[35]

COPD—chronic obstructive pulmonary disease.

## Data Availability

This article is a review and does not report any original data. All data referenced in this work are available through the cited sources.
